# Genetic evidence supports therapeutic hypothesis of increasing progranulin levels for Alzheimer’s Disease

**DOI:** 10.1002/alz.089497

**Published:** 2025-01-09

**Authors:** Chloe Robins, John Eicher, Qin Wang, Jimmy Liu, Dongjing Liu, Thomas Richardson, Jonathan Davitte, David J. Pulford, Padhraig Gormley, Erik D. Ingelsson, Robert A. Scott, Sanjay Kumar, Chun‐ Fang Xu

**Affiliations:** ^1^ Human Genetics & Genomics, Research Technologies, GSK, Upper Providence, PA USA; ^2^ GSK R&D, Cambridge, MA USA; ^3^ GSK R&D, Stevenage, Hertfordshire United Kingdom; ^4^ GSK R&D, Collegeville, PA USA; ^5^ Human Genetics & Genomics, Research Technologies, GSK, Stevenage, Hertfordshire United Kingdom; ^6^ GSK R&D, South San Francisco, CA USA

## Abstract

**Background:**

Genetic variants in *GRN*, the gene encoding progranulin, are causal for or are associated with the risk of multiple neurodegenerative diseases. Modulating progranulin has been considered as a therapeutic strategy for neurodegenerative diseases including Frontotemporal Dementia (FTD) and Alzheimer’s Disease (AD). Here, we integrated genetics with proteomic data to determine the causal human evidence for the therapeutic benefit of modulating progranulin in AD.

**Method:**

Using AD genome‐wide association studies (GWAS) summary statistics, we performed colocalization and Mendelian randomization (MR) analyses with plasma cis‐pQTL data in UK Biobank and CSF pQTL data from neurodegeneration‐focused cohorts. We additionally performed rare variant analyses using UK Biobank whole genome sequencing data to examine the association of *GRN* rare variants with AD and dementia. Finally, we characterized common genetic determinants of progranulin levels genome‐wide in UK Biobank.

**Result:**

We observe consistent supporting genetic evidence that increased progranulin levels associate with reduced AD/dementia risk through GWAS (rs5848, OR = 0.93, p = 2.4e‐20), aggregated rare variant associations (p = 9.18e‐14), pQTL colocalization, and MR evidence. Genome‐wide associations with plasma progranulin levels identify several potential modulators (e.g., *SORT1*, *PSAP*, *APOE*), with *SORT1* showing the strongest common variant association (p<1e‐300). Given the receptor‐ligand relationship between *SORT1* and progranulin, we re‐scaled MR analyses to estimate the therapeutic effect of modulating progranulin with sortilin inhibitors. Our MR analyses suggest that a genetically predicted 2‐fold increase in CSF progranulin levels, as observed in phase 1 clinical trials of a sortilin inhibitor, associates with a ∼46% lower odds of developing AD (OR = 0.54, p = 2.2e‐12).

**Conclusion:**

Our work demonstrates consistent causal associations of *GRN* with AD risk. These data provide causal human evidence supporting the potential therapeutic benefits on AD/dementia of inhibiting sortilin to increase progranulin levels.

